# A singular case of complex suicide by hanging with hesitation marks by axe

**DOI:** 10.1007/s12024-025-00964-2

**Published:** 2025-02-20

**Authors:** Annachiara Vinci, Laura Laura Ambrosi, Marcello Benevento, Davide Ferorelli, Biagio Solarino

**Affiliations:** Section of Legal Medicine, University of Bari, piazza Giulio Cesare, 11, 70124 Bari, Italy

**Keywords:** Forensic science, Hanging, Hesitation marks, Complex suicide

## Abstract

We present a case of complex suicide concerning a 55-year-old man who hanged himself using a rope anchored to a beam on the terrace of his apartment. Multiple parallel linear wounds were observed on his head. At the crime scene, an axe stained with blood and hair was found resting against the wall adjacent to the stairs leading to the upper floor. Forensic investigations identify the cause of death as mechanical asphyxia due to the hanging, in a complex suicide characterized by hesitation marks inflicted on the head with an axe. This specific type of complex suicide has never been described in the literature.

## Case report

A 55-year-old man was found dead on the terrace of his house, hanged with a rope anchored to a beam, with his knees touching the floor and his body facing the wall. Blood stains were observed on the head and hands. On the ground floor, an axe stained with blood and hair was found, along with scattered drops of blood on the stairs leading to the terrace.

**Fig. 1:**
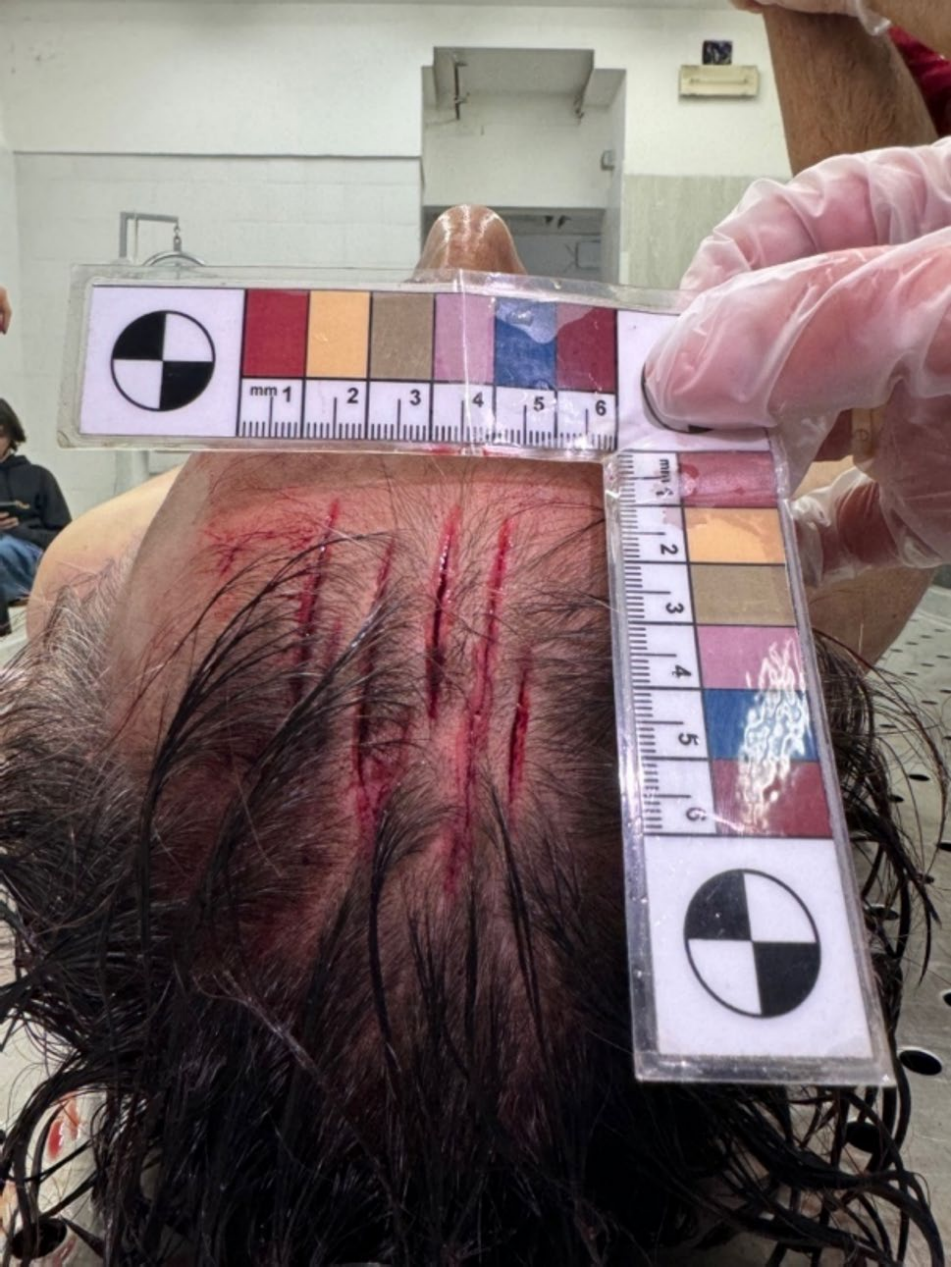
Set of injuries onto the fronto-parietal region of the head

A preliminary external examination revealed conjunctival hyperemia, tongue protrusion clamped between the teeth, a parchment-like skin depression in the cervical region, and spermatorrhea. After cleaning the blood stains, multiple wounds were identified on the fronto-parietal region of the head. This set of injuries consisted of 7 linear wounds within an area of 6 × 4.5 cm, with a predominant anteroposterior axis. They were nearly parallel, varying in length and skin gap, with irregular, infiltrated margins. The wounds lacked fibrous bridging and exposed both subcutaneous and bone tissues beneath (Fig. 1).

**Fig. 2:**
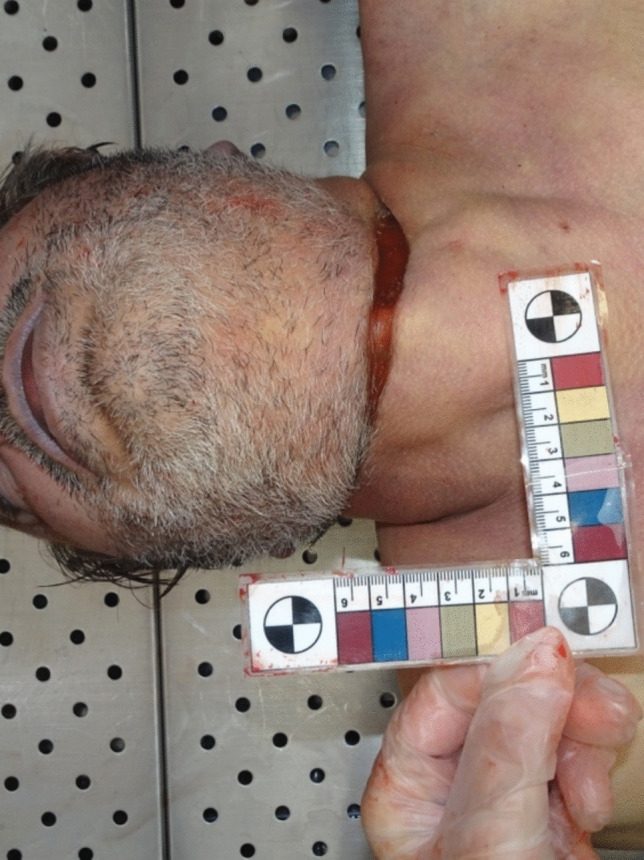
Ligature mark

The neck showed a well-defined, stiff, red-orange, parchment-like ligature mark. The mark was obliquely oriented, extending from top to bottom and latero-medially, measuring 35 cm in length, with an average thickness of 0.5 cm and a depth of 0.4 cm at its deepest point on the left anterior paramedian region of the neck. These findings were consistent with the rope measurements documented during the crime scene investigation (Fig. 2). No defense injuries or additional wounds were identified on other parts of the body.

**Fig. 3:**
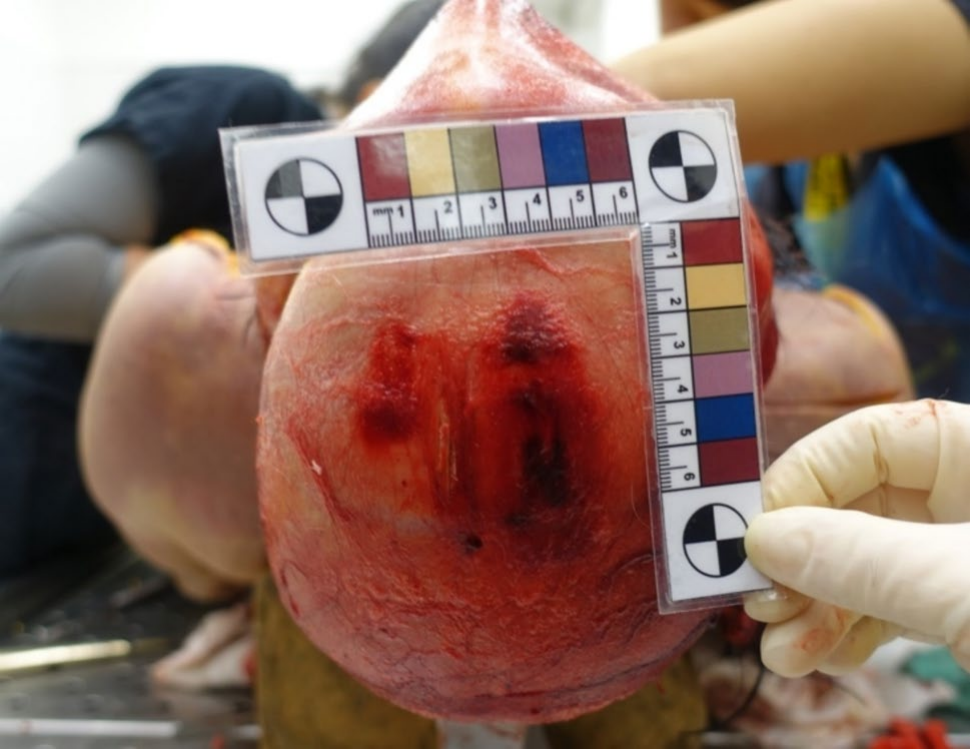
Galea capitis’ hemorrhage

**Fig. 4:**
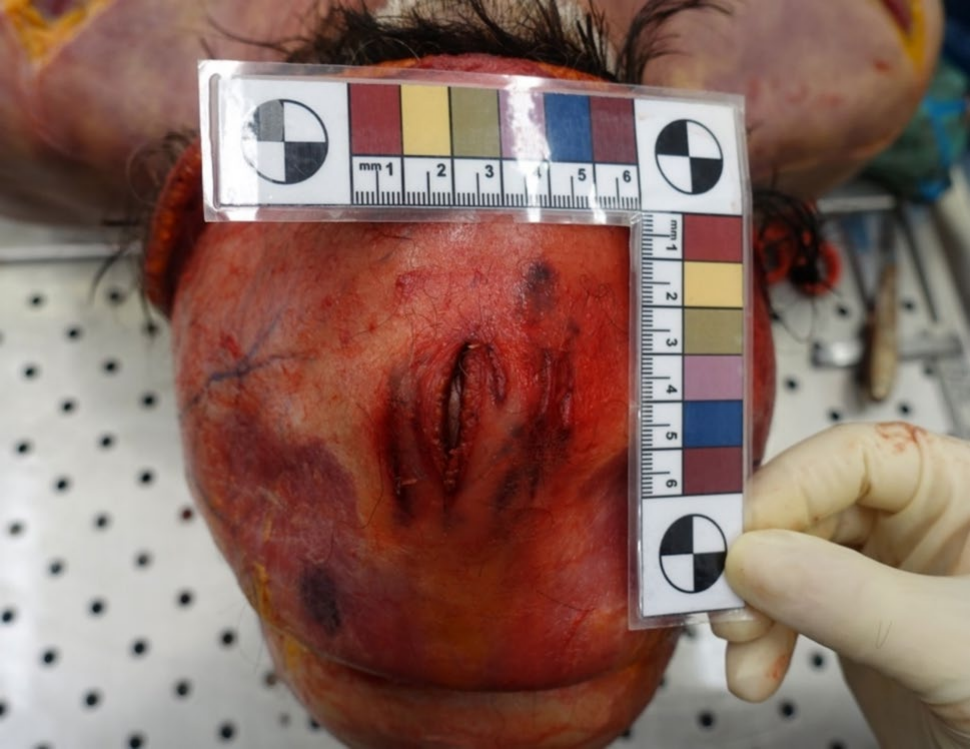
Pericranial subcutaneous tissues’ hemorrhage

**Fig. 5:**
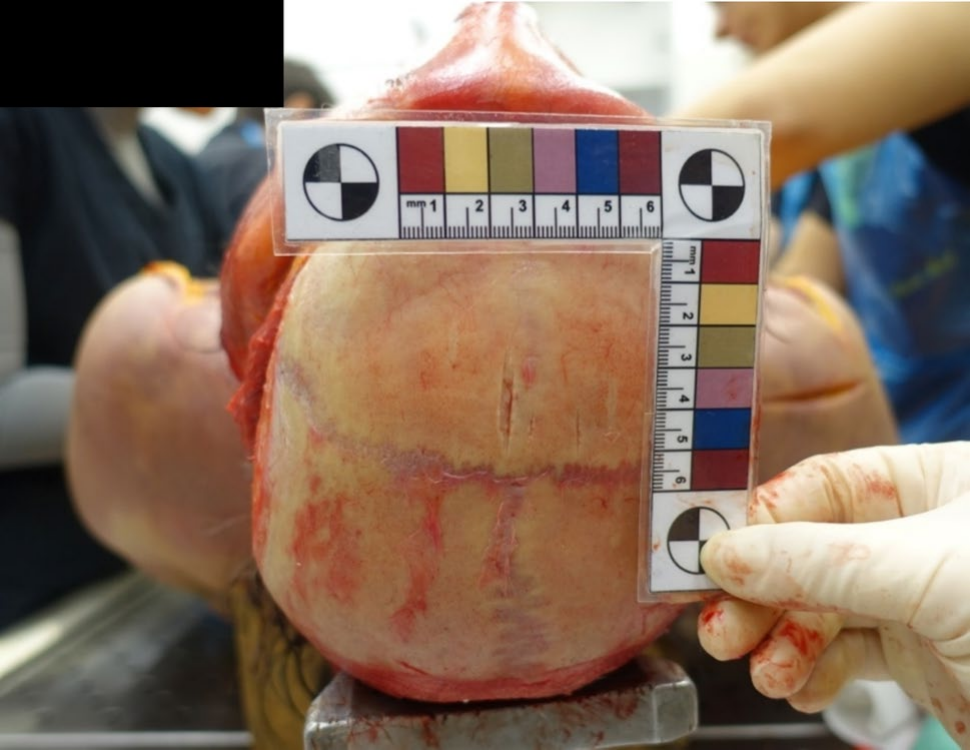
Cranial bone incisions

The autopsy revealed a red-violet, irregularly shaped hemorrhage measuring 5 × 5 cm in median fronto-parietal region of galea capitis, corresponding to the previously described set of injuries (Fig. 3). A similar hemorrhage of 5 × 5 cm in size was also noted in the pericranial subcutaneous tissues (Fig. 4). Upon removal of the periosteum, five linear and parallel bone incisions with variable depth were identified (Fig. 5).

**Fig. 6:**
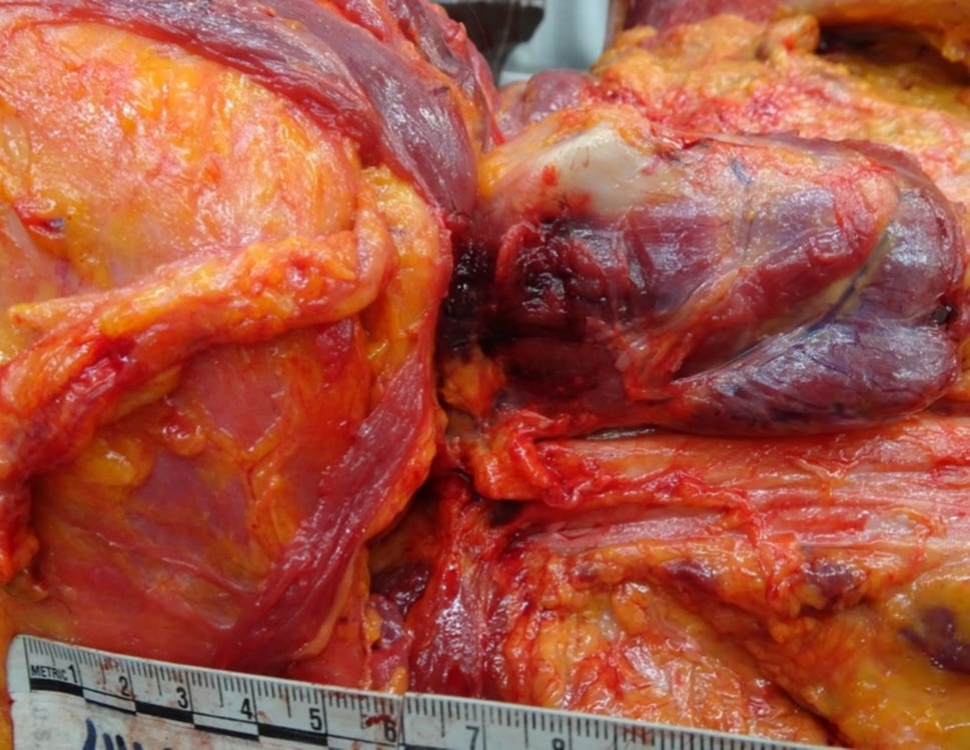
Bilateral hemorrhage of the thyrohyoid muscles

**Fig. 7:**
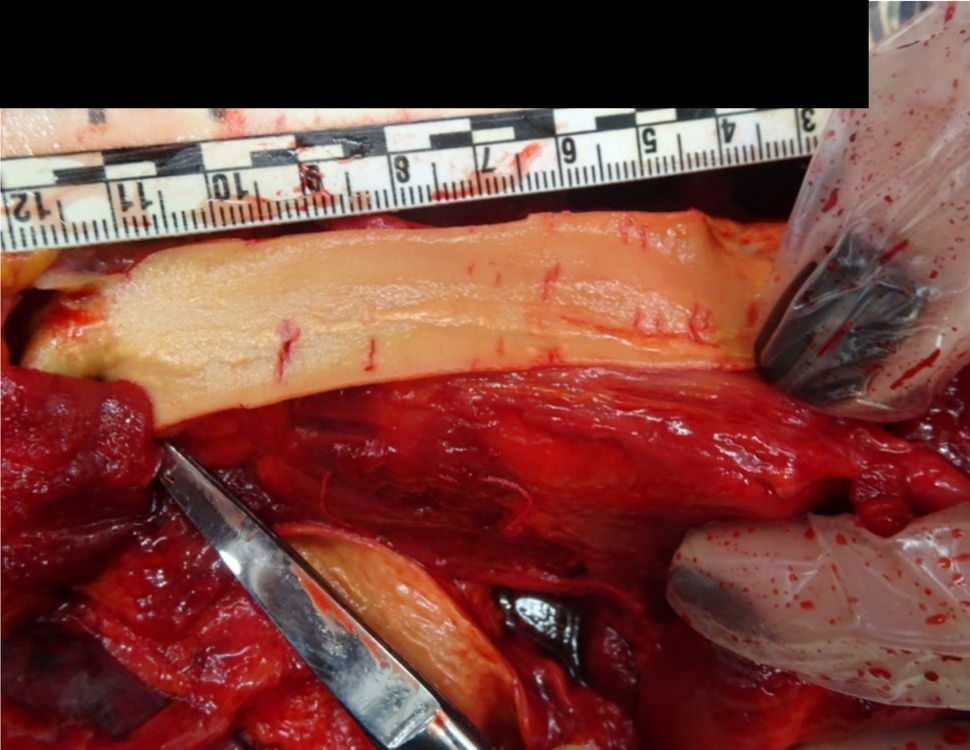
Amussat sign

**Fig. 8:**
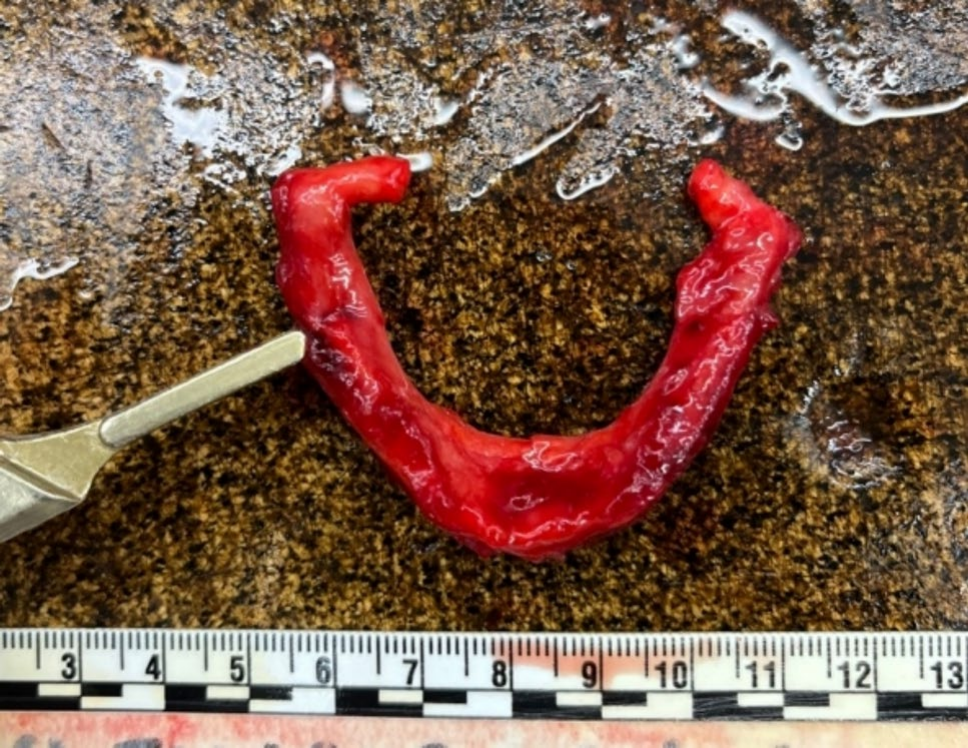
Fracture with associated hemorrhage of the hyoid bone

The autopsy also revealed diffuse poly-visceral congestion, particularly noticeable in the brain, lungs, and liver. Signs of asphyxia included bilateral hemorrhage of the thyrohyoid muscles (Fig. 6), multiple bilateral transverse lacerations of the carotid intima (Amussat sign) (Fig. 7), and the presence of a fracture line with associated hemorrhage at the midpoint of the right greater horn of the hyoid bone were observed (Fig. 8).

Histological analysis further corroborated the widespread polyvisceral congestion and revealed brain hypoxic damage with necrotic neurons and elongated nuclei. Additionally, histological examination of the thyrohyoid muscle and surrounding connective tissue demonstrated extensive hemorrhage.

Findings from the crime scene investigation, external examination, and autopsy, combined with histopathological and toxicological analyses (the latter yielding negative results), established that the cause of death was hanging. The vitality of the ligature mark, the absence of defense injuries, and negative toxicological investigations allowed it to be stated that it was suicide.

**Fig. 9:**
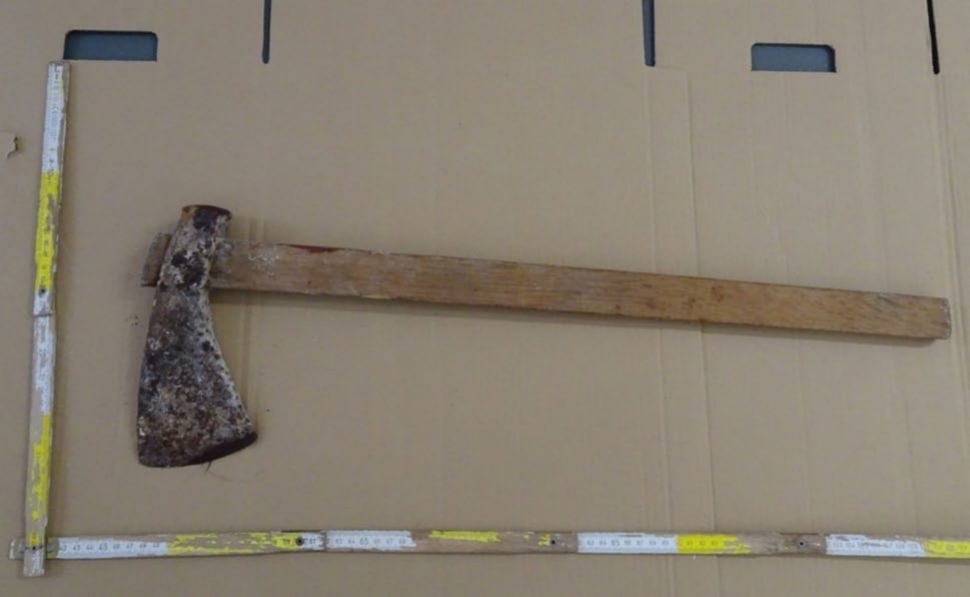
Axe with soiling blood and hair elements on the blade

**Fig. 10:**
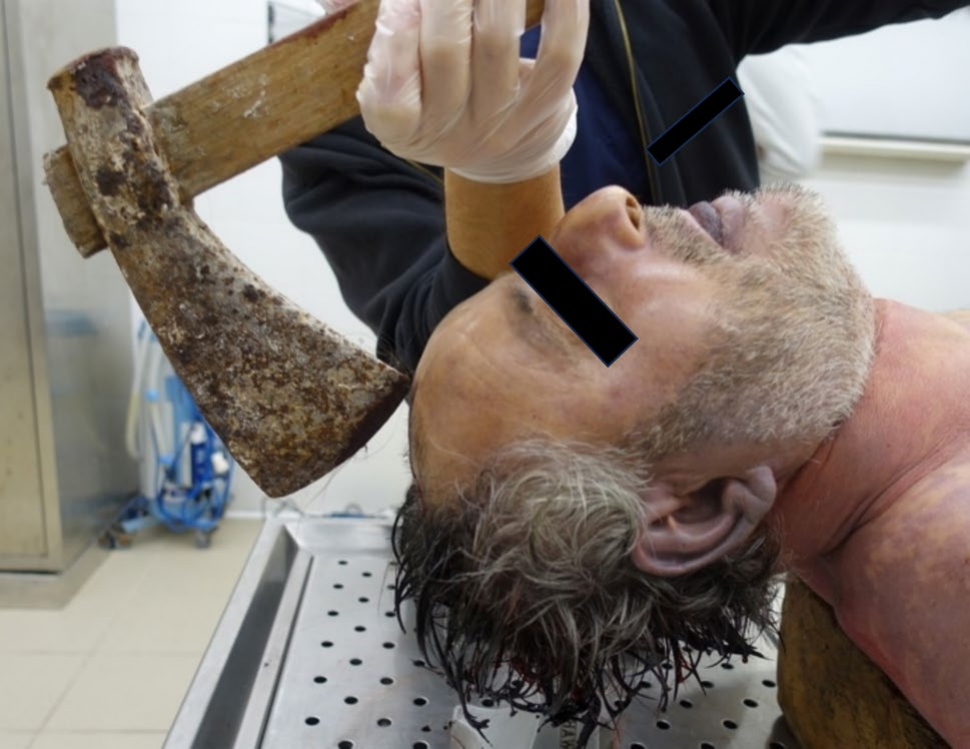
Damaging compatibility between weapon and set of injuries

The axe discovered during judicial inspection weighed 1.7 kg, with a wooden handle measuring 66 cm in length and a cutting blade surface of 9.5 cm. Bloodstains were present on the blade, and bloody handprints on the handle. The blood stain on the cutting surface measured 6 cm in width (Fig.  9). Figure 10 shows how it was possible for the victim to grasp the axe and hit the head (Fig. 10).

## Discussion

Suicide is a prevalent global issue and represents a significant public health concern [1, 2]. The suicide rate among men is more than twice that of women, with older males, particularly those over 60 years of age, being at heightened risk [3]. “Complex suicide” is characterized by the use of multiple self-harming methods to achieve death [4]. According to forensic Literature, complex suicides represent 1.5–5% of all cases of suicide [5]. They are classified into “planned” (or primary) and “unplanned” (or secondary) cases: the former involves the simultaneous application of two or more methods that guarantee the achievement of the death; the latter is characterized by the introduction of a new self-harming method following the failure or ineffectiveness related to a too slow and/or painful lethal potential of the first method applied [6]. Additionally, individuals often select suicide methods based on their availability and accessibility. The most prevalent method of complex suicide involves sharp objects, followed by jumping from a height [3, 7–9].

Moreover, a complex suicide is “primary” if the suicide methods are applied simultaneously and “secondary” if they are applied in sequence [10, 11]. Recently, the term ‘complicated suicide’ has been introduced by some authors to describe cases where a secondary, unexpected fatal trauma follows an initial failed suicide attempt [11]. This scenario complicates the distinction between suicide and accidental death [6]. In unplanned complex suicides, self-inflicted injuries by sharp force, especially cuts to the wrists, are often found as the primary act of suicide. In some cases, the suicide switches from cuts to stabs (mostly to the heart region). Other methods often used after the first phase of suicide are hanging and jumping from a height [12].

Throughout forensic Literature, hesitation wounds are often observed in cases of “complex suicides” due to sharp force injury, and they are significant in the evaluation of the manner of death in each case [13]. Hesitation marks, also called tentative injuries, are superficial cuts or stab wounds in a suicidal context [14]. These are usually multiple parallel cuts of variable depth that indicate repeated trials because of pain or hesitancy before finally cutting through the skin [15]. Though hesitation marks are frequently adjacent to, or overlying, a fatal sharp wound, they are not necessarily near the fatal wounds (e.g., a suicidal victim with a deadly neck cutting and several superficial wrist-cutting lesions as well). The most frequent localizations of hesitation marks are in the neck area, left thoracic area, and wrists. In the upper limbs, hesitation marks are typically found on the flexor surface and radial aspect of the forearm [14].

In determining the cause of death in cases of complex or complicated suicides, forensic pathologists must analyze all available information provided by law enforcement and relatives, conduct a thorough crime scene investigation, and perform a detailed autopsy and toxicological analysis [9].

Complex suicides, including cases involving hanging preceded by stab wounds, are well-documented in the literature [16, 17]. However, this case report is notable for the unconventional weapon employed and the unusual anatomical location of the self-inflicted injuries.

In the present case, the victim’s head exhibited seven linear, non-lethal wounds within an area of 6 × 4.5 cm, with a predominant anteroposterior axis. These wounds were nearly parallel, varied in length and separation, and had infiltrated jagged margins. No fibrous bridging was observed, and subcutaneous and bone tissues were exposed.

In similar cases, distinguishing between hetero-inflicted and self-inflicted injuries can be challenging. The weapon compatible with this pattern of injuries was identified during the judicial inspection. By integrating the historical and circumstantial evidence with the findings from the judicial inspection, external post-mortem examination, and autopsy, it was determined that these wounds were self-inflicted.

This case demonstrates a clear suicidal intent: firstly, the victim inflicted multiple non-lethal wounds on himself with the axe and subsequently ended his life by hanging. The finding of the axe on the ground floor and the dead body on the terrace allows us to affirm that the complex suicide was of the unplanted type. The characteristics of the weapon and the location of the set of injuries suggest that this is a unique case of hesitation marks produced by an axe.

## Data Availability

N/A
